# Recent Advances in Alzheimer’s Disease Research: From Biomarkers to Therapeutic Frontiers

**DOI:** 10.3390/biomedicines12122816

**Published:** 2024-12-11

**Authors:** Raúl López-Antón

**Affiliations:** 1 Department of Psychology and Sociology, University of Zaragoza, 50009 Zaragoza, Spain; rlanton@unizar.es; 2 Centro de Investigación Biomédica en Red de Salud Mental (CIBERSAM), Ministry of Science and Innovation, 28029 Madrid, Spain

At this moment in time, Alzheimer’s disease (AD) remains one of the most pressing public health problems. It is characterized by a progressive and irreversible decline in cognitive function and affects millions of people worldwide. The burden it creates for patients, families and healthcare systems highlights the urgent need for advances in early detection and effective therapies. Although research in this field has grown considerably in recent decades, disease-modifying treatments remain elusive, highlighting the intricate complexity of AD pathophysiology and the need for new diagnostic and therapeutic tools [1,2].

In recent decades, one of the most challenging targets for researchers has been the identification of biomarkers, namely the levels of β-amyloid and phosphorylated tau (p-tau) in cerebrospinal fluid (CSF) and plasma. Recognized as strong indicators of Alzheimer’s disease (AD) pathology, these biomarkers allow for the detection of neurodegenerative changes long before the clinical onset of symptoms. Advances in positron emission tomography (PET) further facilitate the in vivo visualization of amyloid plaques and neurofibrillary tangles, offering transformative tools for the early and accurate diagnosis of AD [3]. These advances in biomarkers not only improve diagnostic accuracy but also enable real-time monitoring of therapeutic efficacy in clinical trials, linking structural changes in the brain to clinical outcomes.

Despite advancements in anti-amyloid and anti-tau therapies, consistent clinical benefits remain elusive. Current treatments primarily provide symptomatic relief and do not halt disease progression, underscoring the critical need to explore alternative therapeutic strategies. The conditional approval of aducanumab represented a historic yet controversial milestone, demonstrating the potential of amyloid-targeted therapies to slow cognitive decline in patients with Alzheimer’s disease (AD) [4]. In this context, the European Medicines Agency (EMA) recently recommended the approval of lecanemab for patients with early AD after re-evaluating its initial decision. This recommendation aligns with the earlier approval granted by the U.S. Food and Drug Administration (FDA). The EMA’s endorsement specifically targets adults with the early symptoms AD who carry one or no copies of the apolipoprotein E4 (*ApoE4*) gene, due to safety concerns associated with other populations. This development, at least temporarily, addresses the ongoing debate surrounding this therapeutic approach [5].

In addition, nanomedicine has emerged as a promising field, promoting innovative drug delivery systems designed to cross the blood–brain barrier, optimizing therapeutic efficacy and minimizing adverse effects. However, the complexity of AD pathology—which includes neuroinflammation, synaptic dysfunction and oxidative stress—continues to challenge efforts to translate these scientific advances into effective clinical treatments [6].

Recent research has highlighted the important role of chronic neuroinflammation in accelerating neurodegenerative processes in AD. Activated microglia, together with proinflammatory cytokines such as IL-1β, IL-6 and TNF-α, contribute to neuronal damage and the progression of neurodegeneration [7]. These insights into immune-related mechanisms open potential avenues for immunomodulatory therapies, and advances in genetic studies are shedding light on other risk factors.

Latest advances in genetic and epigenetic research have revealed important insights into AD susceptibility and progression. Although the *APOE-e4* allele remains an important risk factor, other genetic variants related to neuroinflammation and lipid metabolism have been identified, enriching our understanding of the genetic underpinnings of AD [8]. Epigenetic mechanisms, including DNA methylation and non-coding RNAs, have emerged as critical regulators of gene expression in AD, suggesting potential therapeutic targets. Modulating gene expression through non-coding RNAs represents an opportunity to influence disease progression at the molecular level, opening new avenues for intervention and reinforcing the promises of personalized medicine in AD care.

Addressing AD globally requires coordinated international efforts to address the challenges posed by its complex etiology and regionally varying prevalence. Initiatives such as IDEAL (International Dementia Alliance) and COSMIC (Cohort Studies of Memory in an International Consortium) highlight the importance of adopting culturally and contextually sensitive approaches to AD research. IDEAL, for example, has led to the development of standardized tools, such as the IDEAL Schedule, to assess dementia care needs in diverse populations, facilitating cross-cultural applicability [9]. COSMIC focuses on setting global standards in AD care, especially in low-resource settings where late diagnoses and limited access to care are prevalent [10]. These collaborations emphasize the need to understand epidemiological differences, such as the marked increase in the prevalence of dementia in low- and middle-income countries and highlight the importance of addressing modifiable risk factors to improve global health outcomes [11].

Epidemiological research further highlights the multifactorial nature of dementia, demonstrating interactions between lifestyle, environment and genetic factors. According to the Global Burden of Disease Study, the prevalence of dementia is expected to exceed 152 million cases by 2050, largely due to population aging [12]. Addressing modifiable risk factors—such as physical inactivity, hypertension and smoking- can prevent up to 40% of dementia cases, reinforcing the need for robust public health interventions and creating a basis for preventive approaches in AD research [2]. A fundamental shift in AD research has been the focus on prevention and early intervention, particularly during the preclinical stages of the disease. Advances in biomarkers and genetic profiling now make it possible to identify individuals at high risk for AD long before clinical symptoms appear, facilitating the implementation of preventive strategies ranging from lifestyle modifications to targeted pharmacotherapy [13]. This knowledge highlights the critical role of modifiable risk factors and underlines the value of preventive approaches.

Meanwhile, in the absence of potent disease-modifying drugs, non-pharmacological interventions have shown great promise, particularly in addressing modifiable risk factors in the early stages of AD. Interventions such as cognitive behavioral therapy (CBT), physical activity and structured social support are associated with an improved quality of life, and the evidence suggests that they may also delay disease progression. However, logistical barriers often hinder the implementation of these interventions, particularly in resource-limited healthcare settings where structured programs and trained staff are scarce [1]. Expanding these interventions into routine AD care requires substantial adjustments within healthcare systems, so advances in AI and machine learning (ML) can offer transformative support for predictive diagnosis and personalized treatments.

Neuropsychiatric symptoms, such as depression, anxiety, agitation and psychosis, contribute to accelerated cognitive decline and increase caregiver’s burden, affecting more than 90% of people with dementia [14,15]. Research into the etiology and management of NNS, supported by advances in neuroimaging, has begun to map the specific brain regions associated with these symptoms, offering potential avenues for targeted therapeutic interventions [6]. However, applying non-pharmacological approaches to treating NPS faces similar logistical hurdles to other interventions, especially in resource-limited settings, highlighting the need to develop systemic solutions.

The role of neuroinflammation in the pathogenesis of AD is receiving increasing attention, with the evidence suggesting that chronic inflammation exacerbates neurodegeneration. Immune cells and inflammatory cytokines, such as IL-1β, IL-6 and TNF-α, play a key role in the progression of neurodegenerative changes, making them potential therapeutic targets [6]. Anti-inflammatory and immunomodulatory agents are emerging as promising approaches to modulate AD progression. Future studies are planned to further investigate these pathways, assessing the efficacy of novel immune-targeted therapies in slowing disease progression and paving the way for comprehensive care approaches that reflect the complexities of AD pathophysiology.

Finally, advances in the field of artificial intelligence have created new horizons for research and treatment possibilities. AI models can analyze complex data sets—including neuroimaging, genetic profiles, and biomarker levels—enhancing diagnostic precision and enabling patient stratification using disease risk and progression rates. These technologies hold the potential to identify intricate patterns within data, fostering the development of tailored interventions and significantly improving early diagnostic capabilities [13].

In conclusion, Alzheimer’s disease remains a complex and devastating neurodegenerative disorder that continues to challenge the scientific and medical communities. However, recent advancements in biomarker discovery, genetic research and AI-driven diagnostics lay the foundation for a new era of personalized medicine in AD care. Integrating these scientific insights into clinical practice requires robust interdisciplinary collaboration and dedicated resources to overcome barriers to accessibility and implementation. The future of AD treatment lies in combining scientific rigor with compassionate, multidisciplinary care, translating research advances into tangible benefits for the millions affected by this debilitating disease. While the journey to conquer Alzheimer’s disease is challenging, the rapid strides being made in research, technology and compassionate care inspire hope that, in the not-too-distant future, solutions may be found to preserve memory, dignity, and quality of life for those affected by this illness.
